# Coarse Woody Debris Increases Microbial Community Functional Diversity but not Enzyme Activities in Reclaimed Oil Sands Soils

**DOI:** 10.1371/journal.pone.0143857

**Published:** 2015-11-30

**Authors:** Jin-Hyeob Kwak, Scott X. Chang, M. Anne Naeth, Wolfgang Schaaf

**Affiliations:** 1 Department of Renewable Resources, University of Alberta, Edmonton, Alberta, Canada T6G 2E3; 2 Soil Protection and Recultivation, Brandenburg University of Technology Cottbus-Senftenberg, P. O. Box 101344, 03013 Cottbus, Germany; Leibniz-Institute of Vegetable and Ornamental Crops, GERMANY

## Abstract

Forest floor mineral soil mix (FMM) and peat mineral soil mix (PMM) are cover soils commonly used for upland reclamation post open-pit oil sands mining in northern Alberta, Canada. Coarse woody debris (CWD) can be used to regulate soil temperature and water content, to increase organic matter content, and to create microsites for the establishment of microorganisms and vegetation in upland reclamation. We studied the effects of CWD on soil microbial community level physiological profile (CLPP) and soil enzyme activities in FMM and PMM in a reclaimed landscape in the oil sands. This experiment was conducted with a 2 (FMM vs PMM) × 2 (near CWD vs away from CWD) factorial design with 6 replications. The study plots were established with *Populus tremuloides* (trembling aspen) CWD placed on each plot between November 2007 and February 2008. Soil samples were collected within 5 cm from CWD and more than 100 cm away from CWD in July, August and September 2013 and 2014. Microbial biomass was greater (p<0.05) in FMM than in PMM, in July, and August 2013 and July 2014, and greater (p<0.05) near CWD than away from CWD in FMM in July and August samplings. Soil microbial CLPP differed between FMM and PMM (p<0.01) according to a principal component analysis and CWD changed microbial CLPP in FMM (p<0.05) but not in PMM. Coarse woody debris increased microbial community functional diversity (average well color development in Biolog Ecoplates) in both cover soils (p<0.05) in August and September 2014. Carbon degrading soil enzyme activities were greater in FMM than in PMM (p<0.05) regardless of distance from CWD but were not affected by CWD. Greater microbial biomass and enzyme activities in FMM than in PMM will increase organic matter decomposition and nutrient cycling, improving plant growth. Enhanced microbial community functional diversity by CWD application in upland reclamation has implications for accelerating upland reclamation after oil sands mining.

## Introduction

The Athabasca oil sands region in northern Alberta, Canada, is the largest single oil sands deposit in the world with an estimated 1.6 trillion barrels of bitumen, a low quality crude oil mixed with sands and water [1]. Open-pit mining, one of the most common practices to recover oil from the oil sands in this region [1], has disturbed large areas of mixedwood boreal forests. Oil sands companies are required to reclaim such disturbed lands to equivalent land capability that existed before open-pit mining [2].

A common reclamation practice in this region is returning disturbed land to upland boreal forests. Substrates, such as overburden materials or tailing sands, are not suitable for plant growth due to lack of nutrients, high salinity, and high concentrations of toxic materials including naphthenic acids, polycyclic aromatic hydrocarbons, phenolic compounds and trace metals, and therefore, approximately 30 cm of cover soils are applied over a substrate to support plant growth to supply nutrients and to improve soil properties [3,4]. Soil microbial community and enzymes are essential for organic matter decomposition, nutrient cycling [5–9], and thus plant growth and revegetation [5–9]. Readily decomposable soil organic matter is rapidly consumed by microorganisms, and then decomposition is dominated by turnover of the microbial biomass [5,6]. Microbial community and enzyme activity are important biological indicators of soil quality and net ecosystem productivity in natural and reclaimed ecosystems [7–11]. Characterizing and understanding microbial community and enzyme activity are important for successful land reclamation after oil sands mining.

Soil microbial community and enzyme activities are affected by substrate quality and quantity [9,12,13], plant community composition and productivity [14,15], and abiotic soil properties such as pH, temperature, water content, and aeration [9,11]. Several studies have assessed microbial community structure using phospholipid fatty acids (PLFA) analysis in reclaimed oil sands soils in northern Alberta [11,16–18], and this method mainly measures taxonomic diversity. In addition to taxonomic diversity, knowledge of microbial community function and functional diversity is also important to understand the role of the microbial community in different soils [19,20]. Assessing the soil microbial community level physiological profile (CLPP) is a relatively fast and reliable method for detecting overall changes in microbial community function and structure and Biolog Ecoplates are commonly used to determine microbial CLPP [19,20]. Potential metabolic activity of the microbial community (microbial community functional diversity) is indicated from average well color development in Biolog Ecoplates and community structure based on substrate utilization patterns was assessed with multivariate statistical analyses such as clustering, principal component analysis and canonical correspondence analysis [19,20]. However, the technique has several drawbacks such as culture dependence and the possibility of microbial community growth and change during the incubation [19,20].

Forest floor (litter, fragmented litter, and humus) mineral soil mix (hereafter FMM) and peat mineral soil mix (hereafter PMM) are cover soils commonly used for oil sands reclamation in northern Alberta. Peat mineral soil mix is readily available in northern Alberta and has been used for oil sands reclamation, while the availability of FMM is limited. Applying FMM for oil sands reclamation has recently been used and FMM has several advantages over PMM when used for soil sands reclamation. Properties of cover soils, such as FMM and PMM, used for oil sands reclamation in northern Alberta have been compared [21–24]. The FMM is considered more decomposable with lower carbon to nitrogen (C:N) ratios [24]. Soil water retention is greater in PMM than in FMM due to the higher organic matter content in PMM [17]. As FMM contains more propagules and seeds in seed banks [24], vegetation cover and woody species abundance were greater in FMM than in PMM when the materials were used for land reclamation [23–26]. As soil properties and vegetation covers differ between FMM and PMM when these were applied for reclamation, microbial community and enzyme activities were different in reclaimed oil sands soils [17,18,21].

Coarse woody debris (CWD), including large branches, logs, standing dead trees, and dead coarse roots, plays important ecological roles in forest ecosystems [27,28]. Large amounts of CWD are produced during oil sands mining and they are usually burnt or buried on site. Reclaimed areas are directly exposed to changes in climatic conditions such as temperature, precipitation and wind, due to the lack of vegetation cover. Coarse woody debris plays vital roles in forest ecosystems by regulating soil temperature and water content, controlling soil erosion, increasing soil organic matter content, and promoting spatial heterogeneity and microsites to provide more favorable habitats for microorganisms [27–30] and applying CWD during land reclamation will be beneficial for reforestation. However, only a few studies have evaluated ecological effects of CWD in a reclaimed oil sands landscape [23,25,26].

Many studies assessed the effect of CWD on microbial community and enzyme activities in natural forest ecosystems. Coarse woody debris increased fungal to bacterial ratio, soil respiration, and microbial biomass in temperate coniferous forests [31]. *Gonzalez-Polo et al*. [32] found that CWD application increased C and phosphorus degrading soil enzyme activities in an old growth beech forest in Argentina. However, no study has evaluated the effect of CWD on microbial CLPP and enzyme activities in reclaimed oil sands soils.

This study was conducted to determine if applying CWD on reclaimed oil sands soils amended with FMM or PMM will affect microbial community functional diversity and soil enzyme activities thereby improving soil fertility and accelerating upland reclamation. We hypothesized that 1) soil microbial CLPP would be different between the two cover soils due to their contrasting properties, and microbial biomass would be greater in FMM than in PMM, 2) enzyme activities would be greater in FMM than in PMM regardless of CWD application due to greater microbial biomass and vegetation cover in FMM, 3) CWD would change microbial CLPP due to increased labile C content coming from CWD leachate, and 4) CWD would enhance microbial biomass, average well color development in Biolog Ecoplates, and enzyme activities due to increased availability of microsites. To test these hypotheses, we conducted field experiments 6 and 7 years after land reclamation in an open pit oil sands mining area in the Athabasca oil sands region in northern Alberta.

## Materials and Methods

### Study site

This study was conducted on an oil sands mine site (56° 58’N, 111° 22’W), approximately 24 km north of Fort McMurray, Alberta, Canada. The site was cleared in 1999 for open pit oil sands mining, and thereafter, was used as a saline-sodic overburden waste dump until 2004. The owner of the land (Suncor Energy Inc.) gave permission to conduct the study on this site. A detailed description of the research site and experimental plots is provided in *Brown* [25] and *Brown and Naeth* [23].

**Fig 1:**
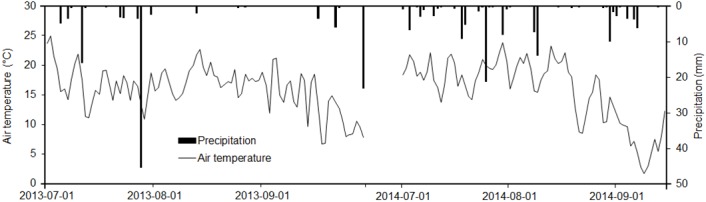
Daily precipitation (bar) and air temperature (line) during sampling periods.

For the study area, average annual temperature from 1981 to 2010 was 1.0°C and average annual precipitation was 418.6 mm, with 316.3 mm as rainfall and 133.8 cm as snowfall [33]. The mean average temperature was 16.0 and 15.1°C for 2013 and 2014, respectively, and the total precipitation during the sampling period, from July to September, was 120.0 mm in 2013 and 154.8 mm in 2014 (Fig 1).

### Experimental design and plot establishment

A factorial experiment consisted of 2 cover soil types (FMM vs PMM) and 2 sampling distances from CWD (near vs away from CWD) with 6 replications was designed for this research. Study plots 10 × 30 m in size were established between November 2007 and February 2008. Six plots were covered with FMM and six plots were covered with PMM. The FMM was applied at a depth of 20 cm on top of 30 cm of B and C horizon mixed subsoil layer over 100 cm of clean overburden. The PMM was applied at a depth of 30 cm over 100 cm of clean overburden [23]. In February 2008, *Populus tremuloides* (trembling aspen) CWD, with diameter bigger than 10 cm, was salvaged and applied on each plot. The CWD pieces did not overlap in the plot to provide maximum contact with the soil surface and covered 10 to 20% of each plot.

Plots were covered by naturally established forbs, grasses, shrubs, and mosses [23,25]. Overall vegetation cover and cover of woody species were greater in FMM than in PMM plots, 65.1 ± 2.1 and 33.9 ± 1.8% per available ground (area without CWD) for FMM and PMM, respectively, and were positively related to CWD cover in the second growing season (2009) after plot establishment [23,25]. Vegetation cover increased over time and remained greater in FMM than in PMM in the fourth (2011) and fifth (2012) years after plot establishment; vegetation cover was similar near CWD and away from CWD; total vegetation covers were 43.4 ± 2.7 and 45.2 ± 1.6% for near CWD and away from CWD, respectively, in FMM and 33.3 ± 3.0 and 30.5 ± 3.4%, respectively, in PMM in 2012 [23,25].

### Soil sampling and analysis

Soil was sampled in the 0 to 10 cm layer using an auger on July 26, August 26, and September 28 in 2013 and on July 8, August 8, and September 4 in 2014. Three soil samples, approximately 30 g each, were collected from each treatment, three within 5 cm from CWD and three more than 100 cm from CWD, in each plot and bulked to form a composite sample of each treatment; a total of 24 composite soil samples were collected [4 treatments (2 cover soils × 2 sampling distance from CWD) × 6 replications] in each sampling. Soil samples were transported to the laboratory on ice packs in a cooler. Fresh soil samples were passed through a 2-mm sieve, and stored in a refrigerator at 4°C until analysis. All analyses were completed within 4 days after sampling. A sub-sample of each soil sample was used to determine available N, microbial biomass C (MBC) and N (MBN), dissolved organic C (DOC) and N (DON), soil microbial community level physiological profile, and extracellular enzyme activities. The remainder of each sample was air dried at room temperature and used to determine pH and electrical conductivity (EC). A portion of the air dried sample was ground into fine powder using a ball mill (MM200, REtsch GmbH, Haan, Germany) and used for total C and total N analyses.

Soil pH was determined using a pH meter (Orion, Thermo Fisher Scientific Inc., Beverly, MA, USA) and EC using an AP75 portable waterproof conductivity/TDS meter (Thermo Fisher Scientific Inc., Waltham, MA, USA) at a 1:5 soil weight to deionized water volume ratio. Available N including ammonium (NH_4_^+^) and nitrate (NO_3_^−^) concentrations were determined via steam distillation [34]. Soil MBC and MBN were determined by chloroform fumigation extraction [35]. Fresh soil samples were fumigated with ethanol free chloroform for 24 hours in an evacuated desiccator. Fumigated and unfumigated samples were extracted with 0.5 mol L^−1^ potassium sulfate solutions at a 1:10 soil weight to potassium sulfate solution volume ratio and filtered using Whatman No. 42 filter papers. Extractable C and N were determined using a TOC-V_CSN_ analyzer (Shimadzu, Kyoto, Japan). To determine DOC and DON concentrations, 5 g of fresh soil samples were extracted with 50 mL of deionized water and filtered using Whatman No. 42 filter papers. Concentrations of C and N in the filtrate were determined using the TOC-V_CSN_ analyzer. Extractable C was used to represent DOC and the difference between extractable N and available N (NH_4_^+^+NO_3_^−^) was used to represent DON. Total C and N were determined using an automated elemental analyzer (NA-1500 series, Carlo Erba, Milan, Italy).

### Soil microbial community level physiological profile

Soil microbial CLPP was determined using a Biolog Ecoplate (Biolog Inc., Hayward, CA, USA), which contains 31 C substrates and one control with 3 replications in a 96 well microplate. One gram of each fresh soil sample was put into a sterile flask with a 100 mL of 0.87% sterilized sodium chloride solution, shaken for 30 min and diluted 1,000 times with 0.87% sterilized sodium chloride solution. A 150 μL aliquot of each soil suspension was inoculated into each well of the Ecoplate. Ecoplates were incubated for 168 hours at 25°C in the dark. The optical density of each well was read at 590 nm every 24 hours using a microplate reader (Emax, Biolog Microstation, CA, USA).

To describe the soil microbial community functional diversity (average well color development in Biolog Ecoplates) and area under the curve (*A*) were calculated with optical density values measured every 24 hours. The average well color development data were calculated according to *Garland and Mills* [36];

Average well color development= ∑(C−R)/n

where *C* is color production within each well, *R* is absorbance of the control well, and *n* is the number of C sources used in the Ecoplate.

The area under the curve *A*_*ik*_ for substrate *i* and plate *k* was calculated by joining the color levels at successive time point of *t(1)*, *t(2)*,…, *t(n)* by straight lines, and summing the areas corresponding to each segment between successive time points by the trapezium rule [37];

$$A=\;\frac{1}{2}\overset{n-1}{\underset{j=1}{\sum }}\left[t\left(j+1\right)-t\left(j\right)\right]\left[C_{ikt\left(j+1\right)}-C_{ikt\left(j\right)}\right]$$

where *C*_*ikt(j)*_ is the color development of substrate *i* for plate *k* at time *t(j)*.

### Soil enzyme activities

Extracellular enzyme activities involved in C and N cycling, including β-1,4-N-acetylglucosaminidase (NAGase, enzyme classification (EC) number 3.2.1.14), β-1,4-glucosidase (EC 3.2.1.21), cellobiohydrolase (EC 3.2.1.91), and peroxidase (EC 1.11.1.x) were measured according to *Sinsabaugh et al*. [38]. Soil sample suspensions were prepared by placing one gram of fresh soil in a 250 mL Nalgene HDPE bottle, adding 125 mL of sodium acetate buffer (50 mmol L^−1^, pH 5), and homogenizing on an end-over-end shaker for 30 min at room temperature. Then soil suspensions were transferred into 96 well plates that were continuously stirred on a stir plate less than 2 minutes to keep soil suspensions homogenized.

For NAGase, β-1,4-glucosidase, and cellobiohydrolase activities, 200 μL of soil suspension and 50 μL of 200 μmol L^−1^ of each substrate were pipetted into black 96 well plates. Reference standards and quench controls were added to each reference and quench well in each plate. The plates were incubated at 20°C in the dark for 3, 3, and 7 hours for NAGase, β-1,4-glucosidase, and cellobiohydrolase, respectively. After the incubation, a 20 μL of 0.5 mol L^−1^ sodium hydroxide solution was added to each well to stop the enzyme reaction. Fluorescence was measured at 360 nm excitation and 460 nm emissions using a multi-detection microplate reader (Synergy HT, Bio-Tek Instruments, Winooski, VT, USA).

For peroxidase activity, 200 μL of soil suspension and 50 μL of 200 μmol L^−1^ of substrate (3,4-dihydroxy-L-phenylalanine) were pipetted onto clean transparent 96 well plates. A 10 μL 0.3% hydrogen peroxide solution was added to each well after the substrate. The plates were incubated at 20°C in the dark for 5 hours. After incubation, absorbance was measured at 460 nm using the multi-detection microplate reader.

### Statistical analyses

Two-way analysis of variance (ANOVA) was conducted to determine the differences in soil properties and each enzyme activity using the SAS software (SAS Institute Inc., NC, USA). Before performing the ANOVA, the normality of distribution and homogeneity of variance were checked with Kolmogorov-Smirnov and Levene’s tests. A repeated measures ANOVA was conducted to assess cover soil type and distance from CWD effects over time on MBC, MBN, each enzyme activity, and average well color development using the PROC MIXED model using the SAS 9.3 software. Distance from CWD was used as a split-plot factor and the month of each sampling was considered a repeated measures variable for determining seasonal variations in 2013 and 2014. In this analysis, the output statistics passed tests for compound symmetry. Tukey’s HSD test was used to determine the significant differences between cover soil type, distance from CWD, month of sampling and their interactions. Pearson correlation and multiple regression analyses were used to determine which soil parameters have strong relationships with MBC, MBN, enzyme activities, and average well color development in Biolog Ecoplates. Slope of changes of average well color development over time was compared using an analysis of covariance.

Color development in each Ecoplate well followed a sigmoidal curve with time and the response of each substrate was different with time; some had short response times while others had longer lags [20]. There was no well leached saturation level before 168-hour incubation and we used average well color development measured at 168-hour for statistical analyses.

The *A*_*ik*_ data were analyzed with a principal component analysis (PCA) to test changes in microbial CLPP as affected by cover soil type and distance from CWD by permutation tests with the Vegan Package of the R software [39]. Statistical differences of microbial CLPP between treatments were assessed using multiple-response permutation procedures (MRPP) [40] with the R software. The significant level was set at α = 0.05 for all statistical analyses.

## Results

### Properties of cover soils

Soil pH, total C, and total N were significantly affected by cover soil type but not by distance from CWD or their interactions, and they were higher in PMM than in FMM (Table 1). Soil EC was significantly greater in PMM than in FMM and greater near CWD than away from CWD in PMM.

**Table 1:** Chemical properties of forest floor mineral soil mix (FMM) and peat mineral soil mix (PMM) collected from near CWD and away from CWD locations in a reclamation experiment.

Treatment		pH	EC^a^	Total C	Total N	C:N^b^
Cover soil	Distance from CWD		(dS m^−1^)	(g kg^−1^)	(g kg^−1^)	
FMM	Near	5.90 (0.12)	0.18 (0.01)	39.7 (4.9)	1.4 (0.2)	30.1 (3.7)
	Away	6.05 (0.10)	0.21 (0.02)	40.8 (5.6)	1.4 (0.3)	32.8 (2.9)
PMM	Near	7.06 (0.03)	0.37 (0.02)	68.4 (8.2)	2.4 (0.3)	28.6 (1.6)
	Away	7.18 (0.04)	0.45 (0.03)	72.7 (10.3)	2.4 (0.4)	31.3 (1.3)
**Two-way ANOVA** ^c^						
Cover soil (S)		***	*	*	*	ns
Distance from CWD (D)		ns	*	ns	ns	ns
S x D		ns	ns	ns	ns	ns

Abbreviations:

^a^EC = electrical conductivity and

^b^C:N = carbon to nitrogen ratio

Values are means with SE (n = 6);

^c^* = p < 0.05;

*** = p < 0.001;

ns = not significant;

Soil DOC concentrations in 2013 were not affected by cover soil type, distance from CWD, or their interactions, except for September 2013 (Table 2). Soil DOC concentrations were significantly greater in PMM than in FMM in July and August 2014. Applying CWD increased DOC concentrations in FMM in September 2014. However, soil DON concentrations were not affected by cover soil type or distance from CWD in 2013 and 2014. Gravimetric soil water contents were significantly higher in PMM than in FMM in July and August but were not affected by distance from CWD in both 2013 and 2014 (Table 2).

**Table 2:** Dissolved organic carbon (DOC) and nitrogen (DON), gravimetric soil water content (SWC), and microbial biomass C (MBC) and N (MBN) in cover soils in 2013 and 2014 and effects of cover soil type (FMM vs PMM) and distance from CWD (near vs away) on soil properties in cover soils used for oil sands reclamation.

Treatment		DOC (mg kg^−1^)			DON (mg kg^−1^)			SWC (%)			MBC (mg kg^−1^)			MBN (mg kg^−1^)		
Cover soil	Distance from CWD	July	Aug.	Sept.	July	Aug.	Sept.	July	Aug.	Sept.	July	Aug.	Sept.	July	Aug.	Sept.
**2013**																
FMM	Near	243.5 (39.8)	302.3 (81.7)	259.8 (23.9)	9.3 (2.0)	8.7 (2.5)	5.4 (1.9)	29.9 (6.9)	25.7 (4.1)	20.6 (4.5)	400.3 (96.0)	382.7 (92.2)	299.7 (65.2)	87.0 (22.9)	65.4 (15.7)	57.8 (11.9)
	Away	220.8 (27.8)	227.4 (21.7)	298.1 (25.4)	7.1 (1.0)	5.7 (0.9)	5.0 (1.5)	26.5 (6.6)	25.0 (5.1)	20.9 (6.7)	267.3 (38.9)	286.6 (39.8)	248.4 (74.0)	55.1 (8.3)	45.7 (10.8)	40.7 (10.1)
PMM	Near	305.8 (26.4)	319.7 (36.0)	323.4 (27.3)	5.6 (1.0)	6.8 (1.1)	2.4 (0.7)	43.2 (6.0)	46.4 (7.2)	32.0 (3.3)	212.1 (44.2)	220.6 (45.5)	279.9 (50.7)	39.9 (7.2)	35.5 (8.2)	37.4 (4.6)
	Away	286.9 (19.1)	332.6 (52.4)	383.7 (38.1)	6.0 (0.9)	7.8 (1.3)	5.1 (1.2)	41.5 (4.6)	47.5 (8.3)	28.2 (4.7)	196.4 (24.6)	173.7 (51.1)	176.4 (35.5)	37.2 (7.0)	33.3 (8.1)	26.1 (6.0)
**Two-way ANOVA** ^a^																
Cover soil (S)		ns	ns	ns	ns	ns	ns	*	*	ns	*	*	ns	*	ns	ns
Distance from CWD (D)		ns	ns	*	ns	ns	ns	ns	ns	ns	*	ns	ns	*	*	**
S x D		ns	ns	ns	ns	ns	ns	ns	ns	ns	ns	ns	ns	ns	ns	ns
**2014**																
FMM	Near	158.7 (25.7)	181.4 (19.3)	150.2 (15.0)	8.6 (0.8)	11.3 (1.7)	8.8 (0.6)	26.1 (4.9)	11.8 (2.6)	20.3 (4.3)	251.8 (52.1)	279.3 (54.6)	347.6 (50.5)	58.2 (11.3)	42.6 (8.3)	59.6 (9.9)
	Away	132.0 (22.6)	146.3 (10.5)	118.0 (7.5)	8.1 (1.8)	10.9 (0.1)	6.8 (0.7)	20.5 (4.7)	10.7 (2.0)	22.0 (2.7)	200.8 (21.1)	250.8 (38.5)	309.9 (54.1)	44.9 (5.8)	34.8 (5.0)	53.2 (8.8)
PMM	Near	192.0 (26.0)	220.4 (21.6)	184.1 (22.6)	7.1 (0.8)	10.2 (1.8)	6.5 (1.0)	38.0 (6.2)	19.6 (2.8)	28.3 (4.6)	144.7 (38.2)	300.3 (57.9)	276.9 (46.3)	35.4 (7.6)	31.7 (6.1)	39.8 (6.9)
	Away	216.1 (23.6)	246.1 (30.1)	176.5 (19.4)	9.7 (1.4)	9.7 (1.1)	6.6 (0.7)	37.9 (5.9)	18.7 (2.2)	32.5 (4.4)	91.5 (28.4)	197.6 (35.1)	237.2 (43.4)	28.3 (6.5)	23.8 (3.5)	33.1 (6.2)
**Two-way ANOVA** ^a^																
Cover soil (S)		*	*	ns	ns	ns	ns	*	*	ns	*	ns	ns	*	ns	*
Distance from CWD (D)		ns	ns	*	ns	ns	ns	ns	ns	ns	*	**	ns	**	**	ns
S x D		ns	ns	ns	ns	ns	ns	ns	ns	ns	ns	ns	ns	ns	ns	ns

Values are mean with SE (n = 18);

^a^* = p < 0.05;

** = p < 0.01;

ns = not significant.

### Microbial biomass C and N

Soil MBC was greater in FMM than in PMM and CWD increased MBC in cover soils; however, effects of cover soils and CWD were variable among sampling months (Table 2). Soil MBC was significantly greater in FMM than in PMM in July and August 2013 and in July 2014, and was greater near CWD than away from CWD in July 2013 and in July and August 2014. Soil MBC was positively related to DOC, DON, MBN, and soil water content in FMM in 2013 and 2014, to DOC in 2013 and 2014 and soil water content in 2013 in PMM (Table 3).

**Table 3:** Pearson correlation coefficient (*r*-value) and significance* among soil variables in cover soils used for oil sands reclamation (n = 36).

Variable^a^	DOC	DON	SWC	MBC	MBN	NAGase	GLU	CEL	PER
**FMM in 2013**									
MBC	0.68**	0.58**	0.78**						
MBN	0.52**	0.60**	0.77**	0.92**					
NAGase	0.52**	-0.08	0.28	0.47*	0.26				
GLU	0.37	0.23	0.66**	0.51**	0.46*	0.53**			
CEL	0.42*	0.29	0.57**	0.58**	0.58**	0.40	0.43*		
PER	-0.11	-0.09	-0.01	0.18	0.15	-0.13	-0.42	-0.05	
**PMM in 2013**									
MBC	0.47**	0.26	0.65**						
MBN	0.36*	0.50**	0.79**	0.79**					
NAGase	0.65**	0.26	0.35*	0.63**	0.48**				
GLU	0.45**	0.50**	0.72**	0.77**	0.79**	0.76**			
CEL	0.38*	0.31	0.29	0.49**	0.54**	0.40	0.54*		
PER	0.01	0.17	0.14	0.09	0.11	0.03	0.22	0.10	
**FMM in 2014**									
MBC	0.68**	0.46**	0.54**						
MBN	0.57**	0.26	0.78**	0.88**					
NAGase	0.84**	0.75**	0.39*	0.64**	0.53**				
GLU	0.69**	0.61**	0.38*	0.48**	0.42*	0.82**			
CEL	0.76**	0.64**	0.60**	0.57**	0.59**	0.84**	0.90**		
PER	0.11	0.07	0.14	-0.12	0.11	0.04	0.24	0.31	
AWCD	0.33*	0.16	0.65**	0.33*	0.51**	0.42*	0.46**	0.54**	0.11
**PMM in 2014**									
MBC	0.31*	0.27	0.28						
MBN	0.38*	0.27	0.72**	0.76**					
NAGase	0.71**	0.72**	0.29	0.47**	0.49**				
GLU	0.63**	0.64**	0.44**	0.44**	0.62**	0.94**			
CEL	0.65**	0.70**	0.30	0.55**	0.56**	0.91**	0.91**		
PER	0.05	0.10	0.26	-0.35	0.09	0.20	0.29	0.13	
AWCD	0.30*	0.27	0.75**	0.11	0.64**	0.45**	0.60**	0.49**	0.53**

^a^Variables: DOC = dissolved organic C, DON = dissolved organic N, MBC = microbial biomass C, MBN = microbial biomass N, SWC = gravimetric soil water content, NAGase = β-1,4-N-acetylglucosaminidase, GLU = β-1,4-glucosidase, CEL = cellobiohydrolase, PER = Peroxidase, and AWCD = average well color development measured after 168 hours of incubation;

Values are Pearson correlation coefficient;

* = p < 0.05;

** = p < 0.01.

Soil MBN showed a similar pattern to that of MBC and was significantly increased by CWD in most samplings, except for September 2014 (Table 2). Soil MBN was significantly greater in FMM than in PMM in July 2013 and in July and September 2014 but was not affected by interaction of cover soil type and distance from CWD. Soil MBN concentrations were positively related to DOC, DON, and soil water content in 2013 and to DOC and soil water content in 2014 in both FMM and PMM (Table 3).

### Soil microbial community level physiological profile and enzyme activities

**Fig 2:**
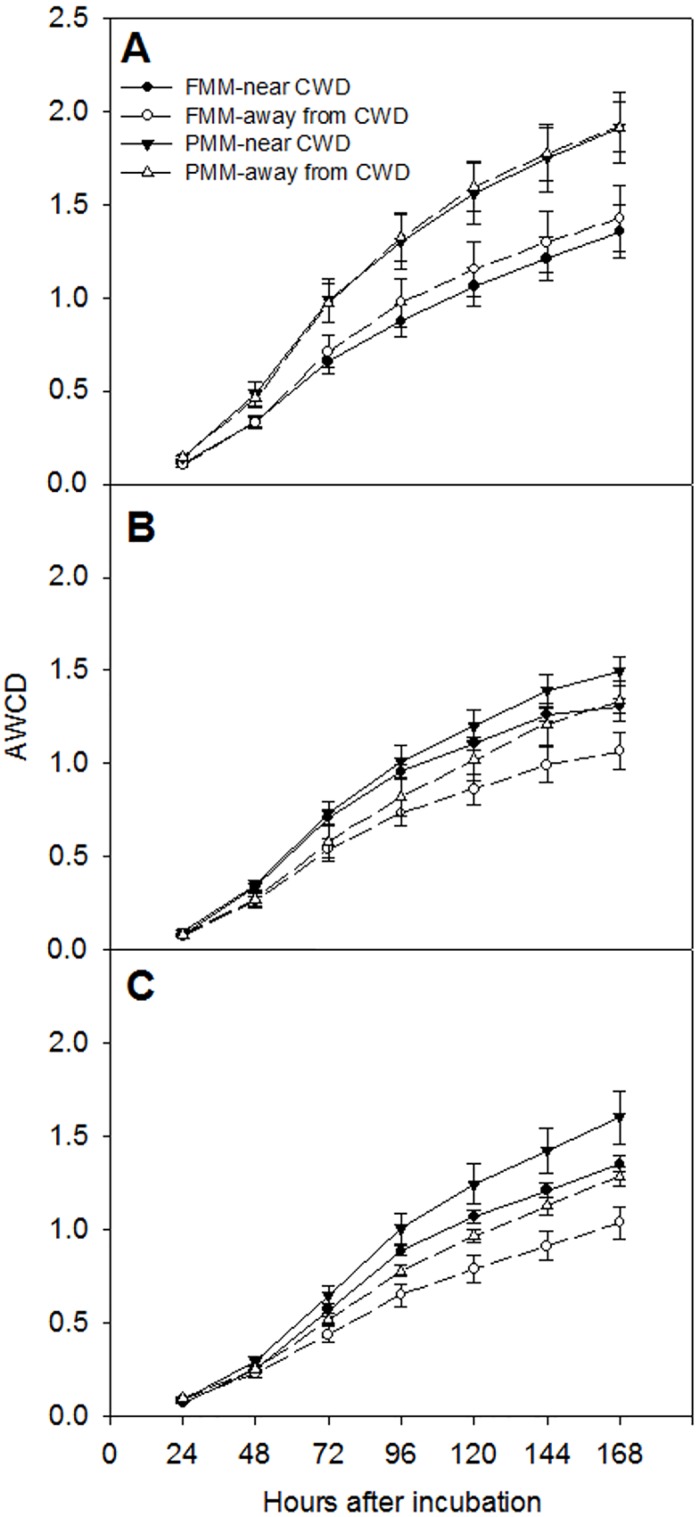
Changes in average well color development (AWCD) in cover soils used for oil sands reclamation; (A) July, (B) August, and (C) September in 2014. Error bars indicate standard errors (n = 6).

Average well color development in Biolog Ecoplates was significantly affected by cover soil type, distance from CWD, and sampling time (Table 4). Average well color development was significantly greater in PMM than in FMM in each sampling regardless of distance from CWD in all sampling times in 2014 (Fig 2). The CWD application enhanced average well color development in August (p<0.001 and p = 0.157 for FMM and PMM, respectively) and September (p<0.001 for both cover soils). Average well color development was positively related to DOC, MBC, and soil water content in FMM and PMM (Table 3). Soil water content and DOC concentrations were the main factors determining the average well color development among treatments, with average well color development = 0.02 × soil water content + 0.001× DOC + 0.814; R^2^ = 0.77; p<0.001; β (standardized coefficient) = 0.71 and 0.15 for soil water content and DOC, respectively.

**Table 4:** Effects of cover soils, distance from the CWD, sampling time and their interactions on soil properties.

Soil properties^a^	Cover soils (S)		Distance from CWD (D)		Sampling time (T)		S × D		S × T		D × T		S × D × T	
	F value	*p* value	F value	*p* value	F value	*p* value	F value	*p* value	F value	*p* value	F value	*p* value	F value	*p* value
**2013**														
MBC	3.13	0.111	7.20	0.025	0.15	0.857	0.14	0.716	1.30	0.286	0.62	0.534	4.00	0.028
MBN	3.67	0.088	13.84	0.005	9.15	0.001	6.44	0.031	1.69	0.199	0.40	0.675	0.95	0.397
NAGase	2.45	0.152	1.41	0.264	77.80	<0.001	0.09	0.768	8.58	0.001	3.06	0.060	0.88	0.424
GLU	9.20	0.014	0.40	0.541	5.24	0.011	0.72	0.419	1.36	0.271	0.00	0.999	0.30	0.745
CEL	10.43	0.010	0.76	0.406	12.25	<0.001	0.05	0.836	2.74	0.078	0.78	0.467	0.72	0.492
PER	7.76	0.013	0.84	0.371	2.09	0.142	2.17	0.159	0.08	0.927	1.94	0.161	0.90	0.418
**2014**														
MBC	1.44	0.258	18.35	0.002	31.32	<0.001	1.47	0.253	5.16	0.010	0.39	0.681	0.75	0.480
MBN	3.15	0.106	15.03	0.003	16.87	<0.001	0.20	0.667	2.42	0.103	0.36	0.701	0.37	0.691
NAGase	3.16	0.106	4.59	0.056	32.66	<0.001	1.62	0.230	0.64	0.531	0.66	0.522	0.59	0.559
GLU	27.66	<0.001	28.17	0.001	49.51	<0.001	6.12	0.035	6.06	0.005	0.45	0.644	0.20	0.817
CEL	17.16	0.002	3.47	0.091	25.62	<0.001	0.61	0.452	2.25	0.119	0.53	0.592	2.53	0.093
PER	46.63	<0.001	0.10	0.765	50.06	<0.001	0.07	0.796	2.29	0.116	3.00	0.062	0.40	0.675
AWCD	6.51	0.029	8.18	0.016	19.49	<0.001	0.01	0.905	3.28	0.049	2.57	0.089	0.34	0.717

^a^Soil properties: MBC = microbial biomass C, MBN = microbial biomass N, NAGase = β-1,4-N-acetylglucosaminidase, GLU = β-1,4-glucosidase, CEL = cellobiohydrolase, PER = Peroxidase, and AWCD = average well color development measured after 168 hours of incubation.

**Fig 3:**
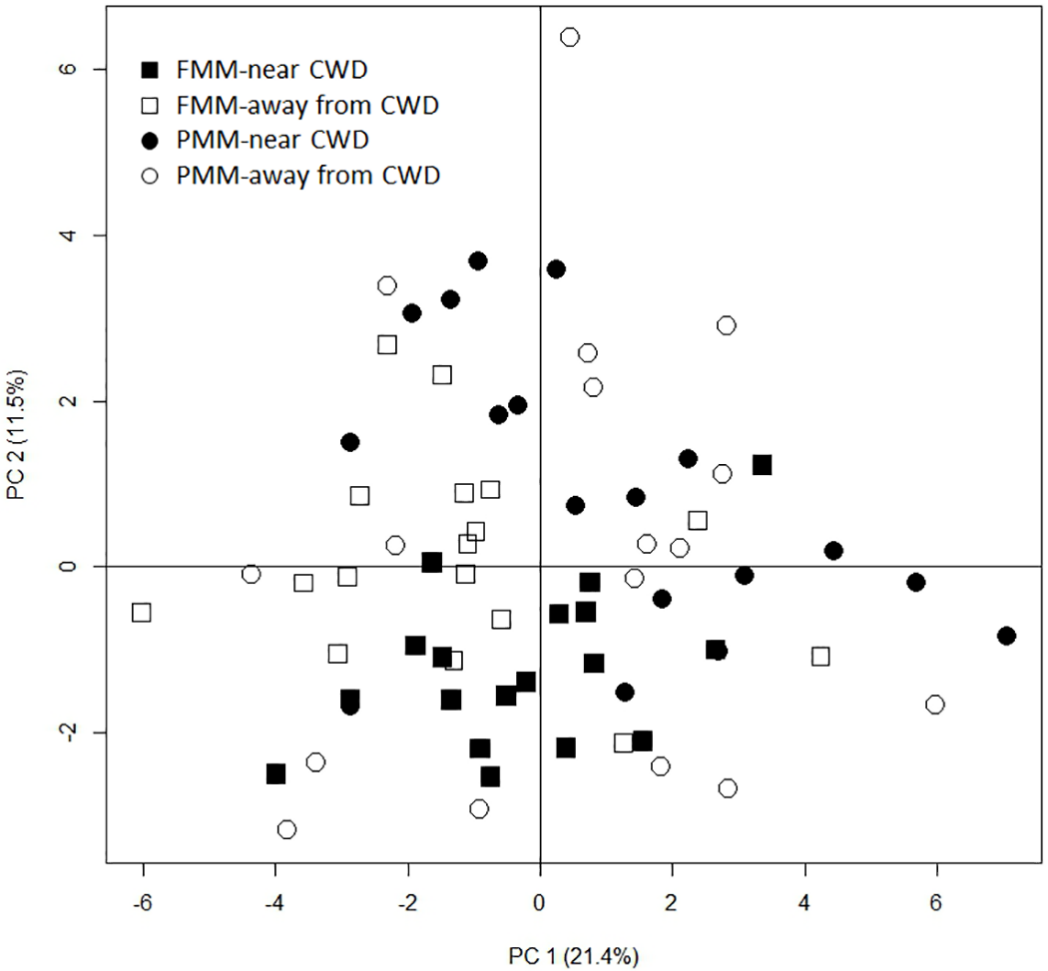
Changes in soil microbial community level physiological profile in cover soils used for soil sands reclamation. Principal component analysis (PCA) of the community level physiological profile in 2014 based on the area under the curve (*A*).

Soil microbial CLPP was different between FMM and PMM (Fig 3, MRPP; p<0.001). The CWD application changed microbial CLPP in FMM (MRPP; p = 0.046) but not in PMM (MRPP; p = 0.124). The first (PC1) and second principal components (PC2) explained 21.4 and 11.5% of the total variation in C substrate utilization profiles, respectively (Fig 3).

**Fig 4:**
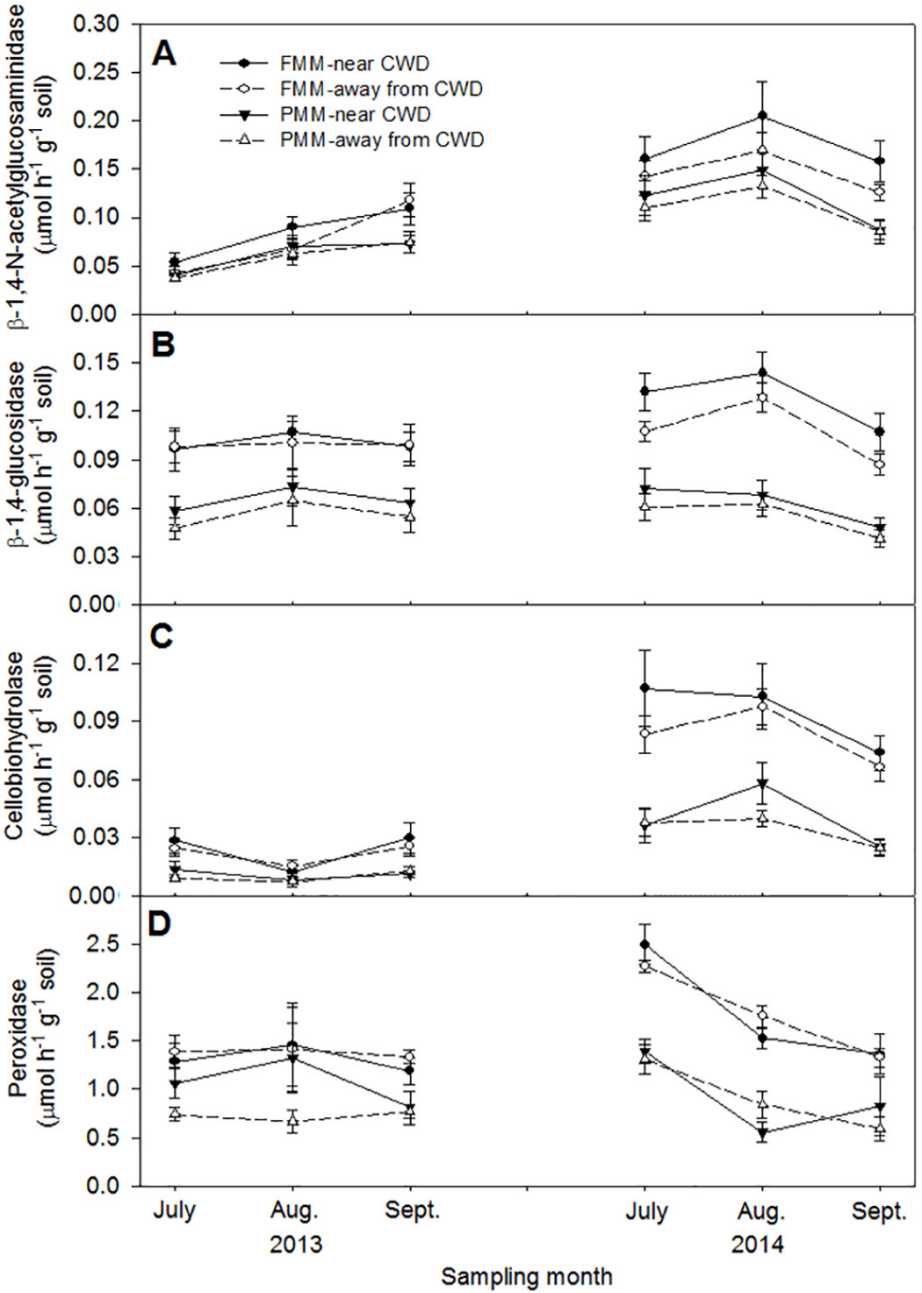
Changes in soil enzyme activities in cover soils in 2013 and 2014; (A) β-1,4-N-acetylglucosaminidase, (B) β-1,4-glucosidase, (C) cellobiohydrolase, and (D) peroxidase activities. Error bars indicate standard errors (n = 6).

Cover soil type and sampling time significantly affected enzyme activities, including β-1,4-glucosidase, cellobiohydrolase and peroxidase in both 2013 and 2014; however, NAGase activities were not affected by cover soil type (Table 4, Fig 4). There was no significant effect of CWD or interaction between cover soil type and distance from CWD on enzyme activities, except β-1,4-glucosidase activity in 2014. Soil enzyme activities in 2014 were significantly greater than those in 2013. Soil enzyme activities were positively related to DOC, DON, MBC, and MBN in both cover soils without any relationship of peroxidase activity with other soil properties (Table 3).

## Discussion

### Soil microbial community level physiological profile and microbial biomass

Soil microbial CLPP in the studied cover soils were significantly different (Fig 3) and microbial biomass in FMM was greater than that in PMM in three out of six samplings (Table 2), supporting part of our first hypothesis. The cover soils, FMM and PMM, used for oil sands reclamation in northern Alberta had contrasting soil properties due to the different sources of organic matter [21–24]. Forest floor is salvaged from upland forests while peat is salvaged from wetlands, causing distinctive microbial community and microbial biomass between FMM and PMM [16,18]. Soil MBC was 4 times greater in FMM than in PMM in the year of plot establishment at the same research site [23]. Although differences in microbial biomass between FMM and PMM were decreased 6 and 7 years after plot establishment, microbial biomass was still generally greater in FMM than in PMM. In addition to initial differences in microbial biomass between cover soils, lower soil pH (Table 1) and soil water content (Table 2) in FMM than in PMM may have caused greater fungal biomass and greater overall microbial biomass in FMM [41] as fungi are favored in acidic soils and more resistant to water stress than bacteria [42]. In our study, however, average well color development in Ecoplates was greater in PMM than in FMM and only some of the bacteria are culturable in the Ecoplate and average well color development represents the function of bacteria [19,20], not fungi. Therefore, greater MBC and MBN but lower average well color development in FMM than in PMM indicates that bacterial biomass was greater in PMM than in FMM while fungal biomass was greater in FMM than in PMM, although we did not directly measure fungal and bacterial biomass.

Both above- and belowground litter and root exudates are primary energy and nutrient sources for soil microorganisms [14,15,43,44] and greater plant litter input from more diverse vegetation cover in FMM [23, 26] likely contributed to the greater microbial biomass in FMM [13]. Greater MBC and MBN in FMM than in PMM in July 2013 and 2014 (Table 2) is likely attributed to greater vegetation cover and release of root exudate in FMM. However, decreases in vegetation productivity and litter input in the later growing season probably caused no differences of microbial biomass in September samplings (Table 2). Greater diversity of plant species in FMM than in PMM [23,26] and contrasting soil properties between two cover soils may cause distinctive microbial CLPP between FMM and PMM [12,15]. Although we did not assess the direct effect of plant community composition on the microbial community, previous studies showed that soil microbial community structure (based on PLFA analysis) was closely related to plant productivity and species composition [14,15,18]. For example, microbial biomass and fungal abundance increased with the higher levels of plant productivity associated with greater plant species diversity in seven years of plant diversity manipulation experiment [15]. Soil microbial community structure was closely associated with plant productivity and community in a Norway spruce (*Picea abies* (L.) Karst.) dominant forest [14] and in reclaimed oil sands soils [18]. In addition, greater mycorrhizal (part of the total fungal community) biomass in FMM than in PMM [25] was associated with higher woody species cover in FMM; such changes may affect fungal to bacterial ratio and soil microbial CLPP.

Applying CWD for land reclamation changed microbial CLPP in FMM but not in PMM (Fig 3), which supported part of our third hypothesis. Increased DOC concentration through CWD leachate in August and September 2014, especially in FMM, which has distinct C composition compared to cover soils [45], may change microbial C utilization pattern and microbial CLPP in FMM [12,13] although CWD had little effect on DOC concentrations. However, CWD did not increase DOC concentrations in PMM without any changes of microbial CLPP. Increases in DOC and MBN in 2014 by CWD, especially in FMM, and positive relationship of average well color development in Ecoplates with DOC and MBN (Table 3) indicates that increased labile C availability by CWD application increased microbial community functional diversity. Greater average well color development in Biolog Ecoplates near CWD than away from CWD in PMM is probably attributed to greater MBC near CWD than away from CWD in 2014 although there was no difference in MBC in September (Table 2). Applying CWD increases microsites [29,30] and provides more favorable habitats for microorganisms and detritivores [27,46]. Increases in detritivore abundance near CWD increases litter fragmentation and can provide more labile substrates for microorganism [46]. Water soluble C or DOC in soils are considered the most readily available C source for soil microorganisms [47,48] and increases in DOC concentration by CWD application may cause positive priming effect [49] and increase microbial community functional diversity. Increased microbial community functional diversity by CWD application suggests that using CWD for oil sands reclamation would enhance nutrient cycling and improve early ecosystem development.

Average well color development in Biolog Ecoplates was strongly affected by soil water content (Table 3) and greater average well color development in July than August or September in PMM is related to the highest precipitation (Fig 1) and soil water content (Table 2) in July. Furthermore, greater soil water content in PMM than in FMM supported more bacterial community resulting in greater average well color development in PMM (Fig 3). Soil water content is a key factor for microbial community function [7]. Bacteria are more susceptible to changing water content [42] and such phenomenon is more significant in reclaimed oil sands soils than in natural soils [18].

### Soil enzyme activities

The greater soil enzyme activities in FMM than in PMM (Fig 4) support our second hypothesis. Soil enzyme activity is affected by soil pH, microbial population size, microbial community structure such as the size of the fungal population size, and plant community composition and productivity [9,21,50]. Lower soil pH and greater woody plant abundance in FMM than in PMM [23,26] increase fungal biomass and mycorrhizal biomass in FMM [7,25,42]. Fungi are responsible for extracellular enzyme production to decompose recalcitrant substrate [51,52], and greater fungal and mycorrhizal biomass in FMM [25] may be linked with greater enzyme activities in FMM. Soil enzyme activities in our study were positively correlated with MBC and MBN, consistent with an earlier study on reclaimed oil sands soils [21]. Higher amounts of litter and root exudates in FMM [26] would increase microbial biomass and enzyme activities [50]. The greater overall enzyme activities in 2014 than in 2013 could also be attributed to increased vegetation cover and litter amount over time [23,25]. Decreases in enzyme activities from August to September in 2014 were probably affected by decreasing vegetation productivity due to decreasing air temperature. Activities of C degrading enzymes such as β-1,4-glucosidase, cellobiohydrolase, and peroxidase, were significantly greater in FMM than in PMM, indicating that organic matter decomposition would be greater in FMM than in PMM. The NAGase activity had strong relationship with N availability [52] and similar NAGase activity in FMM and PMM indicates that N availability would be similar in FMM than in PMM.

We had expected that CWD application would increase soil enzyme activities because of the effect of CWD on vegetation cover and microsites [23]. However, CWD did not affect soil enzyme activities, rejecting part of the fourth hypothesis. Similar soil properties and vegetation cover between near and away from CWD would cause similar enzyme activities between the two locations. As the overall vegetation cover increased over the year in each plot, differences in vegetation cover became smaller between near CWD and away from CWD in later years [26]. The lack of CWD effects on soil enzyme activities were further supported by similar soil properties and vegetation cover between near CWD and away from CWD. A study conducted in an old-growth beech forest showed that C and phosphorus degrading enzyme activities were increased under CWD as compared to soils not under the influence of CWD [32]. In their study, increases in enzyme activities under CWD have been attributed to higher soil moisture content and DOC, and thus increased microbial biomass.

## Conclusions

The two cover soils (FMM and PMM) commonly used for oil sands reclamation had different microbial CLPP, microbial biomass, and enzyme activities associated with contrasting soil properties and vegetation cover originated from different ecosystems. The FMM is a more favorable cover soil for land reclamation due to its greater microbial biomass and enzyme activities relative to PMM, therefore, organic matter decomposition and nutrient supply rates would be greater in FMM than in PMM. The CWD changed soil microbial CLPP in FMM and increased microbial biomass in most samplings and microbial community functional diversity (average well color development in Biolog Ecoplates) in both FMM and PMM. The CWD did not affect enzyme activities, suggesting that enzyme activities were resilient to CWD additions as an additional source of organic matter. Effects of CWD on microbial CLPP and microbial biomass were dependent on cover soil type and sampling time; CWD had greater effects when applied on FMM than on PMM. Increased microbial community functional diversity and microbial biomass by CWD in the studied cover soils suggest that applying CWD for land reclamation would benefit early ecosystem development by increasing organic matter decomposition and nutrient cycling. Applying CWD for land reclamation is recommended for accelerating upland reclamation and for recycling natural resources.
